# Differences among feminist and non-feminist women on weight bias internalization, body image, and disordered eating

**DOI:** 10.1186/s40337-023-00851-7

**Published:** 2023-08-03

**Authors:** Caitlin A. Martin-Wagar, Sarah E. Attaway, Katelyn A. Melcher

**Affiliations:** Department of Psychology, University of Montana, 32 Campus Dr., Missoula, MT USA

**Keywords:** Eating disorders, Weight stigma, Weight bias, Body image, Feminist identity, Feminist beliefs

## Abstract

**Background:**

Research yields mixed results on whether feminist beliefs or self-identification are protective against body image disturbance and eating pathology in non-clinical populations. Further, no studies have examined feminism among those with diagnosed eating disorders. Additionally, previous studies have not examined the relationship between feminist identity and weight stigma. This study investigated these relationships and if there are differences in body image, eating pathology, and weight stigma among feminist identity types in women with eating disorders and college women using ANCOVAs.

**Methods:**

Participants completed self-report measures and were women with eating disorders (*N* = 100) and college women (*N* = 240).

**Results:**

Sixty-four percent of the women with eating disorders and 75.8% of the college women identified as a feminist. An independent samples *t*-test found a significantly higher weight bias internalization in the clinical eating disorder sample than in the college women sample. No significant interactions were found between sample type and feminist identity for body image or weight bias internalization. Results were consistent when using a dichotomous feminist identity item and a seven-item continuous feminist identity item.

**Conclusions:**

Despite the clear impacts of the intersection of weight status and gender, results from this study suggest that identifying as a feminist is not sufficient to combate weight stigma. Findings highlight the need for further research investigating weight bias internalization within eating disorder prevention efforts and interventions.

## Abstract (highlights)

Research shows mixed findings on whether feminist identity is protective against disordered eating and body image concerns. Those with diagnosed eating disorders (EDs) have not been included in previous Research examining the relationship between feminism and the severity of body image symptoms has not included those with diagnosed eating disorders (EDs). Thus, this study examined rates of feminist identity and beliefs in both women with EDs and college women, along with comparing the feminist/non-feminists and ED women/college women on eating disorder-related symptoms. We found that feminist and non-feminist college women and women with EDs did not have different rates of symptom endorsements. Internalization of weight stigmatizing beliefs is known as weight bias internalization (WBI), which means one holds negative beliefs about oneself based on one’s weight. WBI also did not differ between feminists and non-feminists in either sample, but we did find that women with EDs endorsed higher WBI than college women. Given the health implications of weight stigma, our findings highlight the need for further research investigating WBI within ED prevention efforts and interventions.

## Background

Body dissatisfaction in those with eating disorders (EDs) predicts symptom severity, and, for many, body dissatisfaction treatment is key for eating disorder recovery and relapse prevention [25, 43]. EDs are biopsychosocial disorders, with sociocultural factors consistently being potent risk factors for developing and maintaining EDs [10]. One such factor associated with body dissatisfaction and eating pathology is weight stigma and its internalization [14, 31]. Weight stigma is the societal preference for thinner bodies and negative perceptions of higher-weight individuals [40]. Internalization of weight-stigmatizing beliefs is known as weight bias internalization (WBI) and constitutes holding negative attributions about oneself based on weight [13]. Both experiences of weight bias and WBI predict body image disturbance and disordered eating [6].

In contrast, social movements dedicated to dismantling social inequities have the potential to be protective. For instance, feminism, an ideology and a social justice movement that insists women should be afforded the same rights as men in all arenas and rejects the subjugation and discrimination of women [23], has the potential to be useful for those with EDs. Importantly, as noted by Susie Orbach’s groundbreaking anti-diet book *Fat is a Feminist Issue* [35], numerous scholars have asserted that, inherently, fat is a feminist issue and should be treated as such (e.g., [19]. The intersection of gender and weight is notable, Women experience more negative outcomes in a variety of domains related to their combined weight stigma and sexism [19].

A recent ED qualitative study found that 46% of the recovered individuals described feminist-related strategies, such as combating harmful messages and reading feminist writing, as helpful for recovery [46]. Additionally, an older study found that those with EDs differed from other inpatient psychiatric patients in that they had a higher endorsement of feminine gender role stress [32]. However, since this study, gender roles have greatly evolved, and feminism and feminist identity were not examined. Despite the potential relevancy of feminism to combat both sexism and weight stigma, no studies have explicitly examined how feminism relates to ED symptoms and body image in those with diagnosed EDs.

In non-clinical populations, many researchers have found feminist identity and beliefs in women to generally be protective of body image disturbance and the development of EDs (see [33], for a meta-analysis). The beliefs feminists hold, and feminists’ understanding of societal inequities may explain why feminists are more likely to notice that the thin-ideal and Western beauty standards are tools for the oppression of women [33]. Holding sexist beliefs and engaging in the objectification of others predicts oppressive appearance-related practices in women [44]. Women with feminist beliefs would be less likely to hold explicit sexist beliefs or traditional gender role values, potentially protecting them from adverse appearance ideologies or practices.

Though feminism is generally helpful for body image concerns and eating pathology in non-clinical populations of women, the research is mixed depending on the study methodology [33]. Some studies do not find feminist ideology and beliefs to be protective against eating psychopathology [4, 26]. In addition, varied measurements of feminism contribute to conflicting results as to if or how feminism is related to body satisfaction [24]. For example, a study of college women found that the majority endorsed feminist beliefs, but only around 11% labeled themselves a feminist [28]. It can be quite common for people to endorse feminist beliefs, but reject the label of feminist for a variety of reasons, such as stigma with the label or not feeling the feminist movement addresses the saliant needs of some communities [1]. Thus, holding feminist *beliefs* (i.e., holding beliefs that align with feminist ideology) may be a different experience than *identifying* as a feminist (i.e., labeling oneself as a feminist; [4, 24]. Though the methodological differences in studies examining feminism could appear simply semantic, these potentially impact associations and relationships between variables in research [33, 42]. Further, feminism lacks a consistent definition and, therefore, a solid foundation on which to build theory and inform action [12, 23]. Inconsistent conceptualizations of feminism may contribute to why there is inconsistency in the effect of feminism on body image and eating pathology.

Another factor that may impact to how one’s feminism relates to body image and eating pathology is weight stigma. First, there is growing evidence of WBI being particularly impactful for those with EDs. Rates of WBI are significantly higher in treatment-seeking binge-eating disorder (BED) patients with high weight compared to non-eating disordered individuals with high weight [14]. In a transdiagnostic clinical ED sample, researchers found higher levels of WBI related to higher ED symptom severity [31]. Second, weight status intersects with gender. Women face a greater burden related to their combined weight and gender status, even more so for women of color [3]. Higher-weight women experience worse physical and mental health outcomes (e.g., self-esteem, suicide, delayed healthcare resulting in worse health outcomes) than thinner people and men of any size. Despite women facing a disproportionate amount of discrimination based on weight status, weight stigma has received little attention as a feminist issue across feminism [19]. It may be that many people view body size as largely controllable (e.g., “war on obesity”) and one’s personal responsibility, and these beliefs have been found to be linked to more negative outcomes like blame and prejudice [19, 36]. Thus, there are cultural barriers to incorporating anti-weight stigma within some individuals’ conceptualizations of feminist issues.

Further, there are gaps in the research regarding whether feminist identity or beliefs are associated with weight stigma and body image in both clinical and non-clinical populations. With calls to better incorporate an intersectional approach within feminism (e.g., [9], and the pervasiveness of WBI [39], examining weight stigma in relation to women’s feminist beliefs and identity is needed. The act of recognizing weight stigma as a form of oppression requires awareness and education. It is possible that anti-weight stigma beliefs are not always included in conventional feminist belief systems, which may impact whether feminism is protective of body image and eating psychopathology. Indeed, Venturo-Conerly et al. [46] qualitative study on ED recovery, showed how some of the participants identified weight stigma and challenging weight-stigmatizing messages as helpful for their recovery.

There are clear gaps in the literature regarding (1) how feminist identity functions in women with diagnosed EDs and (2) how weight stigma relates to feminist identity and beliefs in any sample. Thus, in the current study, we aimed to examine if there is an interaction effect of sample (ED and college women) and feminist identity on body dissatisfaction and WBI. We hypothesized that only in the college women sample, feminists would have lower body dissatisfaction and WBI than non-feminists. We suspected that in the clinical sample, there would not be significant differences in body dissatisfaction and WBI depending on feminist identity given the more complicated biopsychosocial origins of EDs. We did not expect that feminist self-identification, just one aspect of managing one’s sociocultural environment, would be enough to make an impact the level of body image disturbance or WBI in women with EDs with the other ED biological, psychological, and social risk factors present. Our second aim was to examine differences in WBI between the clinical and non-clinical sample. We hypothesized finding significantly greater WBI in the clinical sample than in the non-clinical sample. For our final aim, we measured the rates of endorsement of feminist beliefs and self-identification. Consistent with prior research, we expected to find a gap between endorsing feminist beliefs and using the label feminist.

## Methods

### Participants and procedure

#### Eating disorder sample

We gathered data as part of a study examining antecedents of eating psychopathology. Participants were 100 women-identifying individuals diagnosed with an ED at specialty ED clinics in the Midwestern United States. Participants needed to be 18 or older, identify as a woman, have an ED diagnosis, and consent to the study. Participants were patients at an eating disorder specialty clinic who had an ED diagnosis determined by an ED specialist clinician using diagnostic criteria from the Diagnostic and Statistical Manual of Mental Disorders—5th Edition [2]. The research team then approached these patients with the option of participating in a voluntary 10–15-min survey. Participants received a five-dollar Amazon gift card for their time. A Midwestern university institutional review board approved all procedures, and we obtained letters of cooperation from the ED specialty clinics before data collection.

An a priori power analysis was conducted using G*Power [18]. For our planned analyses, to detect a small effect size (0.2), with an alpha of 0.01 and power of 0.80, we needed at least 84 participants. We aimed to recruit 110 participants to account for substantial failed validity checks. Two validity items checking for invalid and inattentive responding were included (e.g., choose “rarely”). We removed five participants based on the validity checking, leaving 102 participants. Then, two additional participants only completed the consent form and were removed, leaving a total sample of 100 participants. Participant demographics are in Table 1. Eight (8%) participants did not consent to chart review. As such, we do not have access to their specific ED diagnosis.

**Table 1:** Participant demographics

	Eating disorder sample (*N* = 100)		College student sample (*N* = 240)	
	*n*	%	*n*	%
Race/ethnicity				
White/European American	91	91.0	197	82.1
Asian American	1	1.0	5	2.1
Black/African American	0	0.0	1	0.4
Indigenous/Native American	0	0.0	12	5.0
Middle eastern	0	0.0	1	0.4
Bi/Multi-racial	6	6.0	16	6.7
Hispanic/Latinx	2	2.0	7	2.9
Did not disclose	0	0.0	1	0.4
Sexual orientation				
Asexual	0	0.0	6	2.5
Heterosexual/straight	72	72.0	181	75.4
Lesbian	7	7.0	3	1.3
Bisexual	16	16.0	41	17.1
Other (pansexual, queer, questioning)	5	5.0	9	3.8
Employment Status				
Employed full-time	22	22.0	0	0.0
Employed part-time	15	15.0	0	0.0
Unemployed	17	17.0	0	0.0
On Leave (e.g. FMLA)/disabled	21	21.0	0	0.0
Student	23	23.0	240	100.0
Retired	2	2.0	0	0.0
Eating disorder diagnosis				
Anorexia nervosa/atypical AN	48	48.0	–	–
Bulimia nervosa	7	7.0	–	–
Binge eating disorder	12	12.0	–	–
OSFED	23	23.0	–	–
ARFID	2	2.0	–	–
Missing	8.0	8.0	–	–

#### College student sample

After collecting the data from the clinical sample, we had research questions (outlined in the introduction) that required a non-clinical comparison group. Thus, we then collected a non-clinical college women sample. Participants were 240 women-identifying students at a university in the Western United States. Participants were required to be 18 or older, identify as a woman, and consent to the study. After providing informed consent, participants completed measures via an online survey. We screened out participants who reported having prior ED treatment so we could maintain a non-clinical sample for our comparison group. Participants received partial course credit for their time. The Institutional Review Board approved all procedures at the authors’ institution. Participant demographics are in Table 1.

An a priori power analysis with G*Power indicated that a sample of 120 participants would be needed to detect a small effect size (0.2), with an alpha of 0.01 and power of 0.80. We aimed to recruit 300 participants given the high rates of validity check issues among college populations and our plan to remove those with prior ED treatment. Three validity items checking for attention to the survey questions (e.g., choose “always”) were used to screen out participants who failed the validity items (*N* = 10), leaving a total of 267 participants. Then, for a higher likelihood of a true non-clinical sample, participants who had reported having received ED treatment (*n* = 17. 10.1%) were removed, leaving a total of 240 college women participants.

### Measures

#### Eating disorder pathology

*Eating Disorder Examination Questionnaire* (EDE-Q; [17]) The EDE-Q 6.0 is a 28-item measure used to assess ED behaviors and cognitions during the past four weeks. The measure includes a global scale and four subscales (restraint, eating concern, shape concern, and weight concern). A 7-point Likert scale (0 = *No days*, 6 = *Every day*), with higher scores indicating more ED psychopathology, is used. Sample items include “Have you gone for long periods of time (8 waking hours or more) without eating anything at all in order to influence your shape or weight?” and “Has your weight influenced how you think about (judge) yourself as a person?” McDonald’s omega for the EDE-Q global in this study was found to be 0.85, indicating good reliability [22]. The EDE-Q was used in the ED sample given the clinical utility of the EDE-Q in measuring both ED behavior frequency and ED cognitions corresponding with DSM diagnostic criteria.

*Eating Pathology Symptoms Inventory* (EPSI; [21]. The EPSI is a 45-item measure used to assess eating pathology and attitudes during the past four weeks. The measure includes eight subscales: body dissatisfaction, binge eating, cognitive restraint, purging, restricting, excessive exercise, negative attitudes toward obesity, and muscle building. A 5-point Likert scale (0 = *Never*, 4 = *Very Often*) is used, with higher scores indicating greater ED pathology and more negative attitudes. Sample items include “I did not like how clothes fit the shape of my body” and “I tried to exclude “unhealthy” foods from my diet.” All but one of the subscales demonstrated good reliability, with McDonald’s omega values ranging from 0.80 to 0.91. The muscle building scale was found to have a McDonald’s omega of 0.67, indicating unacceptable reliability, and as such, was not used in analyses. The EPSI was used in the college women sample to measure a wider range of eating pathology that might be seen in a non-clinical population.

#### Body dissatisfaction

*Body Shape Questionnaire *(BSQ; [7]. The BSQ assesses body dissatisfaction. Due to its brevity and sound psychometrics, the 8-item BSQ-8C was used [16, 38]. Questions are on a 6-point Likert scale ranging from 1 (*Never*) to 6 (*Always*), with higher scores indicating more body dissatisfaction. Scores are calculated by summing the eight items. Sample items include (Over the past four weeks…) “Has feeling full (e.g., after eating a large meal) made you feel fat?” and “Have you thought that you are in the shape you are because you lack self-control?” McDonald’s omega for the BSQ-8C was 0.88 for the ED sample and 0.94 for the non-ED sample, indicating good reliability.

#### Weight-based stigma

*Modified Weight Bias Internalization Scale *(WBIS-M; [37]. The 11-item self-report WBIS-M was used to assess one’s level of self-directed weight-based stigma. Sample items include, “I hate myself because of my weight,” “My weight is a major way that I judge my value as a person,” and “Because of my weight, I don’t understand how anyone attractive would want to date me.” McDonald’s omega for the WBIS-M was found to be 0.83 for the ED sample and 0.92 for the non-ED sample, indicating good reliability.

#### Feminist identity

*Cardinal Beliefs of Feminists measure* (CBF; [47]. The CBF was used to examine the endorsement of three basic feminist beliefs: “Girls and women have not been treated as well as boys and men in our society,” “Women and men should be paid equally for the same work,” and “Women’s unpaid work should be more socially valued.” [47]. Participants respond with a dichotomous *Yes* or *No* response to each of the three questions, and “yes” responses are summed. Typically, these three questions are used in concert with a behavioral measure (e.g., answering additional questions if they consider themselves a feminist), but for the purpose of this study, we only examined endorsement of the three basic feminist beliefs, and then asked about explicit feminist self-identification.

*Feminist self-identification and labeling* We examined explicit feminist self-identification in two ways. First, participants selected either *Yes* or *No* in response to the dichotomous question, “Do you consider yourself a feminist?” [29].

**Fig. 1:**
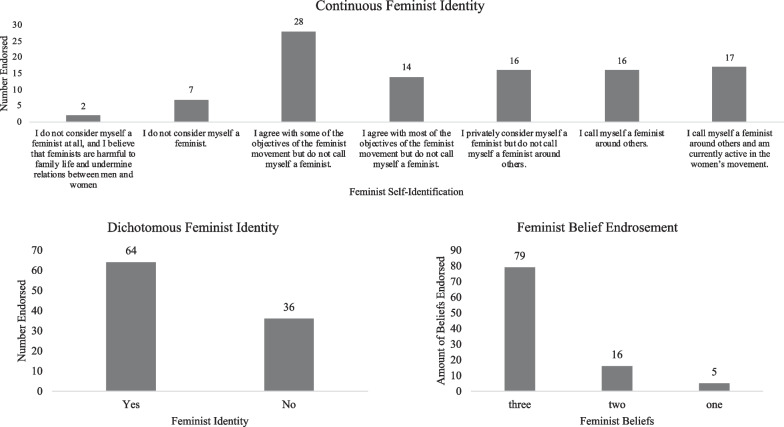
Endorsement of Feminist Identity on a Continuous Scale, dichotomous feminist identity, and feminist belief endorsement (ED sample)

**Fig. 2:**
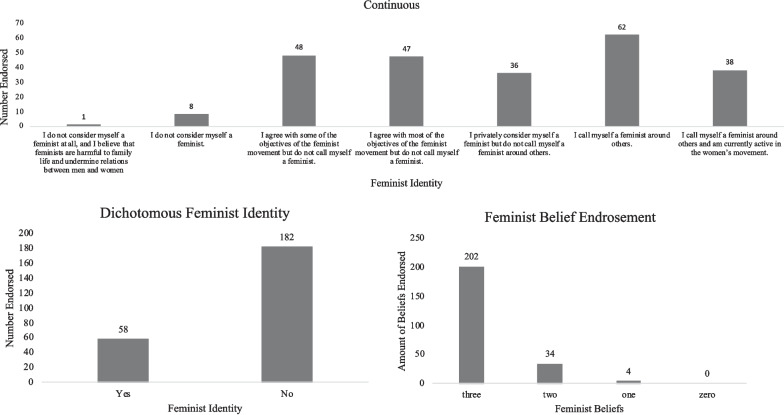
Endorsement of Feminist Identity on a Continuous Scale, dichotomous feminist identity, and feminist belief endorsement (college sample)

Second, a continuous question followed the dichotomous question to determine the degree of feminist identity and self-labeling [34]. Participants chose the self-identification statement that best reflected their self-identification from seven statements ranging from “I do not consider myself a feminist at all, and I believe that feminists are harmful to family life and undermine relations between men and women” to “I call myself a feminist around others and am currently active in the women’s movement” [28, 34], see Figs. 1 and 2).

The dichotomous and continuous questions can precisely determine who self-identifies as a feminist. In contrast, feminist identity stage models or Likert scales with feminist beliefs (and no self-identification) cannot pinpoint who actually identifies with the feminist label.

### Statistical analyses

IBM® SPSS® Statistics 27.0 was used to examine the study hypotheses. First, we computed averages for the study variables and frequencies for feminist beliefs and self-identification (response to the question “Do you consider yourself a feminist?”). We compared the ED to the college sample on study variables with independent samples t-tests, using Cohen’s d for effect size.

Next, we conducted a two-way between-groups analysis of covariance (ANCOVA) to examine the impact of one’s feminist identity (dichotomous yes/no) and sample (eating disorder women/college women) on body image disturbance (BSQ-8C). Then, we conducted another two-way between-groups ANCOVA examining the impact of one’s feminist identity (dichotomous yes/no) and sample (eating disorder women/college women) on WBI (WBIS-M). Finally, given the different levels of feminist identity endorsement on the dichotomous versus continuous feminist self-identification measures, we ran the same ANCOVAs, with the seven-item continuous feminist identity measure instead of the dichotomous item. In each of the two-way between-groups ANCOVAs, we controlled for sexual minority status and age, because previous research has identified these variables as relevant to one’s feminist self-identification. Power analysis for the combined sample analyses suggested a sample of at least 296 participants to detect a small effect size (0.2), with an alpha of 0.01 and power of 0.80.

## Results

Using scale- and subscale-level analyses of missing data, one FBB item was missing in the ED sample, and one individual had missing items on several of the EPSI subscales. Given the low level of missing data (≤ 1%), the missing items were replaced with the mean of the participant’s available items on the respective scales.

### Endorsement of feminist identity and beliefs

For the ED sample, feminist self-identification, as examined by the dichotomous question, “Do you consider yourself a feminist?”, resulted in 64% of the participants indicating *Yes* and 36% indicating *No*. On the continuous feminist identity variable, responses were more nuanced. 78% of the participants endorsed all three feminist beliefs. See Fig. 1. For the college sample, 75.8% of participants considered themselves a feminist, whereas 24.2% did not. 84.2% of the participants endorsed all three feminist beliefs. Similar to the ED sample, responses were more nuanced on the continuous feminist identity variable. See Fig. 2.

### Differences between the ED and college samples

The EDE-Q mean score in the ED sample was 4.13, indicating the participants in this study have ED pathology typical of an ED clinical population [5]. In the college sample, EPSI subscale means ranged from 2.06 (purging) to 15.06 (body dissatisfaction). These scores are within the normal range or below for college student non-clinical populations [20]. Mean body image scores, as measured by the BSQ-8C, were 37.76 (range 8–48) for the ED sample and 26.32 (range 8–48) for the college sample, a statistically significant difference, *t*(338) = 9.45, *p* < 0.001, *d* = 1.19 (large effect), 95% CI [9.06, 13.82]. Average WBI (as measured by the WBIS-M) was 5.09 for women with EDs and 3.70 for college women, a statistically significant difference, *t*(338) = 10.05, *p* < 0.001, *d* = 1.29 (large effect), 95% CI [1.16, 1.63]. See Tables 2 and 3 for descriptive statistics.

**Table 2:** Descriptive statistics on eating psychopathology, body image, and weight bias internalization-eating disorder sample (*N* = 100)

Variable	Total sample		Feminists		Non-feminists	
	*M*	*SD*	*M*	*SD*	*M*	*SD*
EDEQ Global	4.13	1.15	4.04	1.15	4.30	1.13
BSQ	37.76	8.08	38.02	8.00	37.31	8.31
WBIS-M	5.09	0.85	5.13	0.86	5.03	0.84

*BSQ* Body Shape Questionnaire; *WBIS-M* weight bias internalization Scale; *EDEQ* Eating Disorder Examination Questionnaire

**Table 3:** Descriptive statistics on eating psychopathology, body image, and weight bias internalization-college sample (*N* = 240)

Variable	Total sample		Feminists		Non-feminists	
	*M*	*SD*	*M*	*SD*	*M*	*SD*
BSQ	26.32	10.92	26.85	11.37	24.72	9.39
WBIS-M	3.70	1.27	3.83	1.24	3.32	1.33
EPSI NATO	3.56	3.73	3.28	3.48	4.43	4.33
EPSI BE	11.35	7.33	11.71	7.26	10.28	7.56
EPSI CR	4.80	2.89	4.82	2.89	4.76	2.94
EPSI purging	2.06	3.53	2.17	3.81	1.71	2.42
EPSI restricting	8.83	6.04	9.15	5.93	7.96	6.34
EPSI EE	6.79	5.22	6.59	5.14	7.41	5.51

*BSQ* Body Shape Questionnaire; *WBIS-M* Weight Bias Internalization Scale; *EPSI* Eating Pathology Symptom Inventory; *NATO* Negative Attitudes Toward Obesity; *BE* Binge Eating; *CR* Cognitive Restraint; *EE* Excessive Exercise; *MB* Muscle Building

### Interaction testing for sample and feminist identity

In the first two-way between-groups ANCOVA, we examined the influence of feminist identity (dichotomous yes/no) and sample (eating disorder women/college women) on body image disturbance (BSQ-8C), controlling for age and sexual minority status. Levene’s test of equality of error variances was significant (*p* < 0.001), indicating the data violated the homogeneity of variances assumption. Thus, we used a more stringent significant level (*p* < 0.01) for our tests of interaction and main effects, as recommended by Tabachnick and Fidell [45]. There was no significant interaction found between feminist identity and sample (*p* = 0.618). There was a significant main effect for the samples on body image, *F*(1, 334) = 64.92, *p* < 0.001, *Partial η*^*2*^ = 0.163 (large effect size). No main effect was found for feminist identity on body image, *F*(1, 334) = 0.79, *p* = 0.376, *Partial η*^*2*^ = 0.002. When conducting the ANCOVA with the seven-item feminist item instead of the dichotomous item, the same significant relationships (and lack of relationships) remained.

In the next two-way between-groups ANCOVA, we examined the influence of feminist identity (dichotomous yes/no) and sample (eating disorder women/college women) on WBI (WBIS-M), controlling for age and sexual minority status. Levene’s test of equality of error variances was significant (*p* < 0.001), indicating the data violated the homogeneity of variances assumption. Thus, we again used a more stringent significant level (*p* < 0.01) for our tests of interaction and main effects, as recommended by Tabachnick and Fidell [45]. There was no significant interaction found between feminist identity and sample (*p* = 0.184). There was a significant main effect for the samples on WBI, *F*(1, 334) = 82.65, *p* < 0.001, *Partial η*^*2*^ = 0.198 (large effect size). No main effect was found for feminist identity on WBI, *F*(1, 334) = 2.95, *p* = 0.087, *Partial η*^*2*^ = 0.009. Again, when conducting the ANCOVA with the seven-item feminist item instead of the dichotomous item, the same significant relationships (and lack of relationships) remained.

## Discussion

In line with our hypothesis and previous research [28, 33], we found that feminist belief endorsement did not necessarily translate to feminist self-identification. Approximately eight in ten individuals endorsed all three feminist belief items in both samples. However, only approximately two-thirds to three-fourths of the participants used the label feminist. When examined more specifically, only 33.0–41.7% of the participants indicated they would call themselves a feminist around others. In our study, a larger proportion of the samples endorsed calling themselves a feminist than in previous studies, yet belief endorsement and self-identification remained inconsistent. Findings are potentially related to younger generations having less stigma attached to the feminist label, prominent social movements like #MeToo, corporation endorsement of feminist messaging, and high-profile celebrities and politicians using the feminist label (e.g., [15]. Previous research and our findings emphasize a systemic and pervasive difference between endorsing feminist beliefs and self-identification, highlighting potential stigma, misunderstanding, or inclusivity issues related to the feminist label [1, 24]. Additionally, this disparity between the endorsement of feminist beliefs and feminist self-identification stresses theoretical and methodological issues related to the empirical examination of feminism [42]. Future research might benefit from examining the best methodological approaches that are sensitive to changing contexts, and how and why one’s social identities may impact endorsing feminist beliefs and using the feminist label.

As hypothesized, in the ED sample, feminists did not significantly differ from non-feminists on body image and WBI. However, contrary to our hypothesis, in college women there were also no significant differences between feminists and non-feminist on body image and WBI. Despite the clear impacts of the intersection of weight status and gender [3], it may be that identifying as a feminist is not sufficient for combating internalizing weight stigmatizing messages and overwhelming pressure for femme-appearing people to be thin. Combating weight stigma to prevent internalization may be a separate skill from combating other gender-related messages. Of note, feminist scholars have provided long-standing criticism of the lack of a commonly agreed-upon definition of feminism [12, 23]. Thus, individuals identifying as feminists may include anti-weight bias attitudes within their feminist framework to varying degrees. For example, feminism has been critiqued for its lack of recognition of intersecting identities [8, 23], and some expressions of feminism have even perpetuated other systems of oppression, such as homophobia and racism [11]. Even if anti-weight stigmatizing attitudes are part of one’s feminist identity, weight stigma may stem from several other assumptions, such as the link between weight and health. Despite evidence that contradicts the direct link between health and weight or body size (e.g., [30]), the current societal emphasis placed on this perceived link continues to perpetuate weight stigma.

The finding that even in the college sample, feminist and non-feminist women did not significantly differ in body image and eating pathology differs from some of the previous research [33]. However, as noted, the research on the relationship between feminist identity and body image and eating pathology has been variable, with other studies finding no relationship between feminism and eating pathology in non-clinical samples [4, 26]. The feminist movement, priorities, and membership have also deeply changed over time [41]. Further, the recent increase in postfeminist ideologies and individuals identifying as feminists may create new associations with the identity, highlighting the need for new research examining feminist identity across ED-related variables. However, having only one definition of feminism may never be feasible or helpful. Intersectional feminism, for example, includes recognition of how other privileged or marginalized identities interact and combine with gender identity to create power and oppression [8]. Future theory and research should qualitatively examine the overlap (and lack thereof) between being feminist and anti-weight-biased. Lack of intersectionality has been described within feminism [8], and, unsurprisingly, being a feminist does not necessarily mean one is also anti-weight biased, just as being feminist does not mean one is anti-racist. Future examination of intersectional feminism should include anti-weight bias, which is particularly important in women with EDs, who often have high WBI and body image disturbance.

Our findings do not indicate that tenants of feminism (such as identifying from where messages about appearance are coming and challenging those messages) are not relevant to all with EDs. Venturo-Conerly et al. [46] found that 46% of recovered individuals cited feminist-related strategies as helpful during recovery. Clearly, how feminism operates within clinical EDs is poorly understood and in its infancy. Research examining if teaching specific skills in line with feminism and fat liberation are helpful within ED treatment is needed, especially given the high rates of WBI in ED populations [14, 31]. Consistent with previous research comparing a higher-weight BED sample to a non-clinical higher-weight sample [14], in our study, women with EDs had significantly higher WBI than college women. Perhaps interventions that explicitly include anti-weight bias techniques could be examined within ED treatment.

Several limitations and strengths are worthy of discussion. First, given that this study was cross-sectional, it is unknown if feminism impacts recovery from an ED or how feminism might relate to preventing EDs for individuals who would have otherwise developed an ED. There is also a restricted range for the study variables in the clinical ED sample, so it is unsurprising that the vast majority would have high rates of eating pathology and body image concerns. Thus, longitudinal and qualitative studies examining the role of feminist identity throughout the treatment process could highlight where, if anywhere, feminism may be useful within treatment. This sample of women with EDs was also majority White. Recruitment occurred in insurance-funded treatment centers that typically have a lower percentage of women of color due to inequitable access to care. Treatment studies using grants may help increase sample diversity by the mechanism of explicitly searching for representative samples of women with EDs to include in the study. As a strength, our study was able to remove college students who had previously received ED treatment from our sample to better ensure a non-clinical comparison group. Further, this study is the first to examine WBI and feminist identity together. We also examined feminist identity in relation to eating pathology and body image in a clinical sample of women with EDs for the first time. Finally, we were able to compare a transdiagnostic ED sample to a college non-clinical sample on their WBI.

## Conclusions

The findings from this study contribute knowledge about feminism and WBI within clinical ED populations and a non-clinical at-risk population (college women). For women with EDs and college women, feminists and non-feminists did not differ in the severity of symptoms. The finding that ED women having higher WBI than college women, addressing WBI in prevention and intervention efforts is needed. With ED treatments being insufficient (e.g., [27] in helping a majority of those with EDs reach recovery, a better understanding of treatment targets is vital, and addressing weight stigma and its internalization are approaches that hold potential within ED treatment.

## Data Availability

The datasets generated and/or analysed during the current study are available in the Open Science Framework repository, https://osf.io/r9d7n/?view_only=4452bb51008a49d094404cdd20c46f57.

## Abbreviations

APA: American Psychiatric Association
BED: Binge-eating disorder
BSQ: Body Shape Questionnaire
CBF: Cardinal beliefs of feminists
ED: Eating disorder
EDs: Eating disorders
EDE-Q: Eating Disorder Examination Questionnaire
EPSI: Eating pathology symptoms inventory
ANCOVA: Analysis of covariance
WBI: Weight bias internalization
WBIS-M: Modified Weight Bias Internalization Scale
